# Bacteria isolates from Lake sediments as a promising proxy for temporally tracking organic pollution: A global review^☆^

**DOI:** 10.1016/j.onehlt.2025.101184

**Published:** 2025-08-30

**Authors:** Julie O'Donovan, Jean O'Dwyer, Michelle McKeown, Aaron Potito

**Affiliations:** a Palaeoenvironmental Research Unit, School of Geography and Archaeology, University of Galway, Galway, Ireland; b Irish Centre for Research in Applied Geosciences, University College Dublin, Dublin, Ireland; c School of Biological, Earth and Environmental Sciences, University College Cork, Cork, Ireland.; d Department of Geography, University College Cork, Cork, Ireland; e Environmental Research Institute, University College Cork, Cork, Ireland; f Co-Centre for Climate, Biodiversity and Water, United States of America

**Keywords:** Lake sediment Core, Faecal Indicator Bacteria (FIB), *Escherichia coli*, *Enterococci*, Antimicrobial resistance (AMR), Organic pollution, Faecal contamination

## Abstract

Growing populations, agricultural intensification and inadequate wastewater treatment have led to rising organic pollution in surface water bodies, posing significant public health risks, particularly from waterborne pathogens. Faecal indicator bacteria (FIB) are widely used to monitor water quality and detect organic pollution in water bodies. This systematic review was undertaken to identify and synthesise existing literature on how FIB can be used to track historical changes in organic pollution in lake sediment records and to identify key research gaps. The review summarises prevalent bacterial indicators and existing methods used to analyse how trends in bacterial abundance relate to pollution both spatially and temporally. Beyond bacteria, this review also focuses on the prevalence of antimicrobial resistance (AMR) in sediment records. Overall, key environmental variables affecting bacterial distribution and persistence include organic content, metals and sediment grain size. Particular attention must be given to these variables in future studies for a clear understanding of temporal bacteria distribution in sediment records. The review also highlights the importance of accurately dated sediment cores and historical pollution context to correlate and interpret bacterial distribution patterns over time. Gaps in the literature were observed with only two studies tracking the changes of AMR over time, and many of the existing studies limited to the same lake. The findings of this review emphasize the need for more robust future research across multiple lakes and exploring AMR profiles in sediment cores to understand the evolution of resistance through time.

## Highlights

• A global scoping review on the ability of bacteria in lake sediment cores to track organic pollution over time.
• Few number of studies identified after inclusion and exclusion criteria were applied (*n* = 12)
• *E*. *coli* and *Enterococcus* were the most widely used faecal indicator bacteria to track pollution in the sediment cores.
• Factors that may affect bacteria and AMR distribution and persistence include organic content, metals and grain size.
• Future research focuses: monitoring AMR in sediment cores and conducting more comprehensive studies across multiple lakes.

## Graphical abstract

**Figure:**
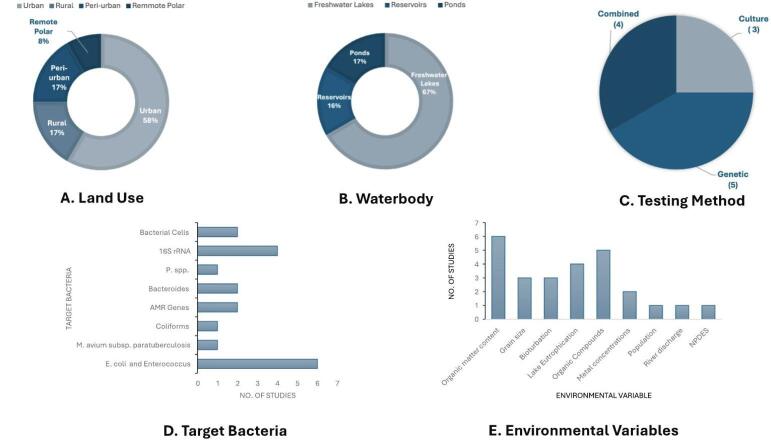

## 1 Introduction

The management of surface waters remains a key challenge due to anthropogenic influence, with eutrophication, acidification and contamination primary drivers of surface water pollution, impacting both ecological and human health [1,2]. While clean drinking water, which is typically derived from freshwater sources (i.e., both surface water and groundwater) and treated to remove contaminants, is vital to human health, the freshwater resources themselves are susceptible to contamination by waterborne pathogens; thus, posing a serious threat to human health if effective water treatment and monitoring systems are not employed [3]. Exposure to waterborne pathogens in domestic water supplies can cause gastrointestinal disease, increasing the pressure on healthcare systems, particularly affecting children, the elderly and the immunocompromised [[4], [5], [6], [7]]. In 2019, it was estimated that insufficient water, sanitation and hygiene (WASH) could be attributable to 1.4 million deaths and 74 million disability-adjusted life years (DALYs) globally [8]. For children under five years old, this accounted for 7.6 % of all deaths and 7.5 % of all DALYs globally [8]. According to Wolf (2023), in 2019, the majority of the safe drinking water, sanitation and hygiene (WASH) -attributable burden was due to diarrhoeal disease, accounting for more than one million deaths and approximately 55 million DALYs. The main cause of diarrhoeal disease is pathogen ingestion via the faecal-oral route from sources such as contaminated drinking water [9]. As such, the management of wastewater is paramount to prevent gastrointestinal disease at a global scale. However, data collected from 140 countries and territories in 2022 indicates that only 58 % of domestic wastewater flow was safely treated [10]. Moreover, recent studies suggest antimicrobial-resistant infections have increased the burden of enteritis via two key point sources: wastewater treatment plants and drinking water supplies [11].

Further, faecal pollution of waterbodies can result in the development of antimicrobial resistance (AMR) in the environment over time, posing additional risks to those exposed and adding to healthcare costs due to the need for more intensive and costly treatment options [11,12]. Faecal indicator bacteria such as *Escherichia coli* (*E. coli*) can become resistant to certain antibiotics as it is exposed to antibiotics ingested by the host in the intestine it inhabits [13]. AMR is one of the biggest known threats to public health globally [14], highlighting the importance of understanding the prevalence of microbial pollution and AMR in waterbodies which may be utilised by humans.

Faecal indicator bacteria (FIB) are key indicators of faecal pollution in surface waters and domestic water supplies [15]. FIB such as *E. coli* or *Enterococcus*, which can be released into the environment through wastewater discharge and in faeces such as runoff from farmlands, have routinely been used to assess levels of organic pollution in aquatic systems [16,17]. The amount of faecal pathogenic bacteria in streams notably increases when livestock, specifically cattle, have direct access to waterbodies [18]. FIB have been known to accumulate in freshwater sediments as the bacteria become attached via adsorption to sediment particles in the water column, which then settle and build up over time, becoming a part of the sediment's microbiome [18,19]. Importantly, a range of environmental parameters (e.g., pH, temperature) and mechanisms/processes (e.g., hydrophobicity, biofilm formation) can influence the attachment ability of bacteria to sediment particles in freshwater environments [20]. Specifically, *E. coli* can persist in lake sediments, which can act as environmental reservoirs for faecal bacteria in freshwater habitats, and are incorporated into the sediment profile [21]. The abundance of *E. coli* in the water column can also increase due to the transition of *E. coli* into the water from sediment during resuspension [22]. In addition to resuspension, post-depositional processes such as bioturbation, where sediment can be reworked by benthic organisms, can redistribute bacteria vertically within the sediment column [23]. This can potentially disrupt the chronological resolution, making it more difficult to reconstruct historical pollution patterns.

Given the ability of FIB, particularly *E. coli*, to persist and accumulate within freshwater environments, lake sediments offer a valuable archive for assessing historical trends in faecal contamination. Paleolimnology, the study of lake sediments to reconstruct past environments, has provided insights into long-term environmental change for nearly two centuries [[24], [25], [26]]. To gain retrospective (temporal) insights into anthropogenic pollution in freshwater environments, paleoenvironmental datasets can be constructed and consulted [27,28]. The paleolimnological tool-kit enables the temporal reconstruction of past physicochemical and biological statuses of aquatic systems via the analysis of indicators incorporated and preserved in the sediment record [[26], [27], [28]]. Indicators in lake sediment cores have been used to reconstruct historic eutrophication [[29], [30], [31], [32]], metal pollution [28,32,33], and organic pollutants [34,35]. Specifically in relation to FIB, some studies have used faecal biomarkers, such as faecal stanols or sterols in sediment cores taken in aquatic environments, as indicators or ‘proxies’ to track faecal pollution through time [36,37]. Despite these advances in proxy development, a key limitation in applying FIB to sediment-based reconstructions is their limited preservation potential. Unlike other biological proxies which can be well preserved and applied as paleo-indicators, FIB do not readily fossilise, and as such, time pressure from the time of deposition can become an issue in their application in paleoenvironmental studies [[38], [39], [40]].

Gaining a deeper understanding of the prevalence and sources of pathogens such as faecal bacteria is essential for guiding future research and informing policy decisions aimed at protecting the environment and safeguarding public health. To date, no comprehensive global literature review has specifically examined faecal pollution through isolating FIB from freshwater sediment cores. Previous research employing FIB has primarily focused on classical depositional environments such as lake and river surface sediments [41] and marine sediment cores [42]. While aquifers [43]are not traditional depositional environments, they have been investigated as subsurface microbial habitats. However, relatively few studies have focused on temporal changes in freshwater lake sediments using FIB as a proxy. This represents a key knowledge gap for tracking faecal pollution and its potential to spread pathogenic diseases across spatial and temporal scales. As such, the presented study aims to conduct an extensive scoping review of the international peer-reviewed literature to identify, collate, and critically assess whether bacterial isolates from lake sediment cores can reliably indicate historical faecal pollution trends and to explore the environmental conditions affecting their preservation and detectability in sediments. This review also aims to highlight methodological approaches used across studies, identify key knowledge gaps and evaluate the limited research on AMR in sediment cores. In addition, key environmental parameters such as sediment organic content, grain size and metal concentrations are examined for their influence on bacterial preservation and distribution over time. While spatial aspects are occasionally discussed where relevant to multiple lake sediment cores taken in investigations, the primary emphasis of this review is in the temporal application of these bacteria isolates to assess historical pollution. In doing so the study design reflects an effort to inform and refine future paleoenvironmental investigations into organic and microbial pollution using bacterial indicators.

## 2 Methods

This review focused on evaluating the use of bacterial isolates, ARGs and key environmental parameters such as organic matter, grain size and metal concentrations as indicators of historical faecal pollution in freshwater lake sediment cores. These parameters were chosen based on their relevance in influencing bacterial persistence, detectability and association with anthropogenic pollution inputs.

### 2.1 Primary research question, bibliographic database searches and literature identification

**Fig. 1:**
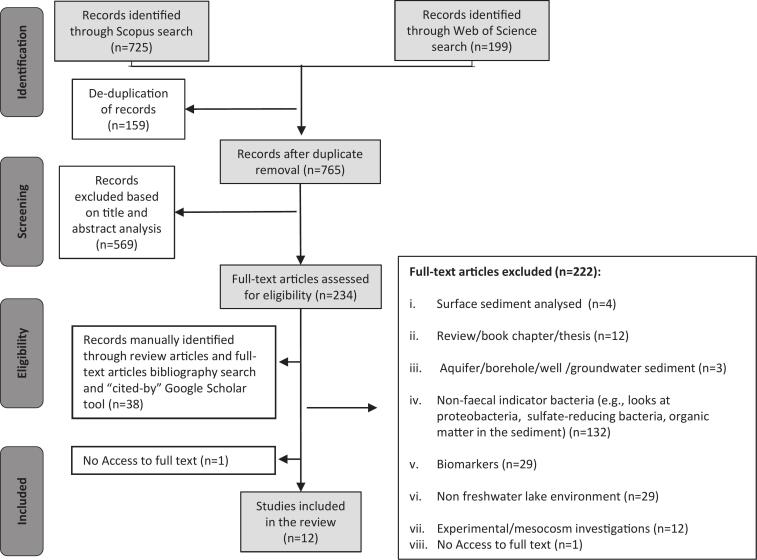
Flow chart of the protocol used to complete the literature review showing the key steps (Identification, Screening, Eligibility) ultimately leading to the final selection of articles (Included) in the study.

The literature identification protocol used in this scoping review (Fig. 1.) was based on methodologies adapted from several key sources, following established guidelines [[44], [45], [46], [47], [48], [49]]. This approach facilitated a structured and comprehensive search for relevant studies. The primary research question was formulated to direct and refine the literature search process in the scoping review:

*“What is the prevalence and persistence of faecal indicator bacteria (FIB) in lake sediment cores and what (if any) are the potential applications in tracking organic pollution in lake systems through time.”*

The criteria used for search term development and different categories employed in the literature search are provided in Table 1. Literature searches were confined to two bibliographic databases, Scopus and Web of Science, for the identification of suitable studies. A modified version of the Population-Outcome-Agent (POA) model [50] was used to develop search terms enabling a series of ‘mock’ searches in the two databases. The finalised search terms employed in the review are provided (See Table S1, Supplemental Material), with database searches first conducted in October 2022 and updated searches completed in June 2025. Boolean positional operators (“AND” and “OR”) were applied to all database searches to filter the results. For Web of Science, the field tag “TS” was applied to the search to narrow the search terms to the topics of the articles, while in Scopus, the field tag “TITLE-ABS-KEY” was applied to the search to target the titles, abstracts, and keywords.

**Table 1:** Search term development used in database searches and their classifications.

Term Classification/Theme	Description	Search terms
Population: Freshwater sediment cores	Environmental population of interest	“Pond Core”, “Lake Core”, “Lagoon Core”, “Reservoir Core”, “Freshwater Core”, “Inland water core”, “sediment Core”, “Lake Core”, “Lentic Core”, “Lake Substrate Core”, “Lake Sediment Core”, “Lake Core”, “Lake Transect”, “Lake Stratigraphy”, “Lentic Core”
Agent and Consequence: Bacteria	Organism(s) of interest and relevant effect on environmental population	“faecal”, “faecal”, “faecal indicator”, “faecal indicator”, “faecal indicator organism”, “faecal indicator organism”, “FIO”, “coliform”, “coliforms”, “total coliforms”, “total coliform” OR “faecal indicator bacteria”, “faecal indicator bacteria”, “FIB”, “coliphages”, “coliphage” OR “microbial indicator” OR “gram-negative bacteria” OR “gram-positive bacteria”, “bacteria”, “microbial”, “E.coli”, “*E. coli*”, “*escherichia coli*”, “enteric bacteria”, “enterococcus”, “enterococci”, “microbe”
Receptor/ Consequence/ Outcome:	Relevant effects on environmental population	contamina* OR pollut* OR presence OR incidence OR preserv* OR dissemination OR dispersal

#### 2.1.1 Literature screening and study selection

Overall, a total of 924 studies were originally identified with de-duplication resulting in 765 studies (Table 2; Fig. 1). The articles were then screened for suitability based on their title and abstract by applying an established set of inclusion and exclusion criteria (Table 3). As a result, articles which did not meet the eligibility criteria outlined in Table 3 were excluded from the review, leaving 196 studies for full-text screening. Articles that had no full text available (*n* = 1) were excluded from the review.

**Table 2:** Total number of studies retrieved from each database and final count after duplicate removal (via Endnote).

Identified Records	924
Scopus	725
Web of Science	199
Duplicate articles removed	159
**Records after duplicate removal**	**765**

**Table 3:** Inclusion/exclusion eligibility criteria employed for literature screening.

**Inclusion Criteria**	**Exclusion Criteria**
**Study type:** All primary research articles (peer reviewed), short communications.	**Study type:** Academic reviews, grey literature, book chapters, conference proceedings.
**Language:** English	**Language:** non**-**English
**Population:** Sediment cores from freshwater aquatic environments (i.e. lakes, ponds, reservoirs) with focus a on faecal pollution.	**Population:** Surface sediments, sediments from non-aquatic environments, marine sediment cores, river sediment cores, marsh sediment cores, floodplains, aquifer cores.
**Agent:** Any bacteria (e.g., *E. coli*) which indicates faecal pollution.	**Agent:** Analysis of only indicators or proxies other than bacteria which indicates faecal pollution, broad DNA analysis of all bacteria profiles present in the environment (e.g., sulfate reducing, cyano bacteria)
**Study design:** Environmental or palaeoecological investigations.	**Study design:** Meta-analytical investigations; laboratory-based or controlled experimental settings.
**Period:** Any - present	

Criteria applied in article screening included: i) all primary research articles and short communications that were peer-reviewed, ii) articles published in English, iii) studies based on sediment cores from freshwater aquatic environments (i.e., lakes, ponds, reservoirs), iv) analysis of bacteria (e.g., *E. coli*) which indicates faecal pollution in core samples from relevant sources, and v) investigations based on environmental or palaeoecological study designs. Studies were excluded for the following reasons: i) non-original peer-reviewed articles such as academic reviews, literature reviews, conference proceedings, grey literature, or book chapters, ii) non-English articles, iii) investigations based on surface sediments, non-aquatic environments (e.g., soil profiles), or marine sediment cores, river sediment cores, marsh sediment cores, floodplains or aquifer cores iv) articles which only analyse indicators or proxies other than bacteria which indicates faecal pollution (e.g., nitrogen cycling bacteria, firmicutes), broad DNA analysis of all bacteria profiles present in the environment (e.g., sulphate reducing bacteria, cyanobacteria) and v) any meta-analytical investigations, laboratory-based investigations or articles based on research carried out in controlled experimental settings.

#### 2.1.2 Additional literature identification

Supplementary manual searches were completed during November 2023. Review articles identified during the initial screening process were analysed to identify any relevant studies, which may not have been captured in the database searches (*n* = 14). By manually screening the bibliographies of articles that met the eligibility criteria (Table 3), seven additional studies were identified. Articles selected for full-text screening were further screened using the “cited by” tool in Google Scholar to identify any subsequent studies which may have cited the article. This identified articles potentially overlooked in the database search, leading to the identification of 38 studies. The established inclusion and exclusion criteria were then applied to these additionally identified articles, resulting in six of these studies meeting the criteria and being included in the final total of 12 studies.

### 2.2 Data extraction

Data extraction and organisation efforts were based on an MS Excel spreadsheet. Data fields were categorised under 10 main categories (See Excel Table S2, Supplemental Material). A total of 53 data fields were extracted from each study. “Not reported (NR)” was applied to data fields when there was no extractable information available or the data was ambiguously reported in the study. Only data pertinent to sediment core collection in freshwater aquatic environments (i.e., lakes, ponds, reservoirs) were included from each study in this review. Particular attention was paid to extracting data on core parameter of interest including the presence and abundance of faecal related bacteria and ARGs and environmental variables such as sediment organic content, grain size and metal concentrations. These variables were selected based on their known influence on the survival and transport of faecal bacteria in aquatic sediments and their relevance in tracking organic pollution through time. Studies which did not report on these parameters were excluded unless they contributed essential information regarding the research question. Where reported, data on bacterial survival, persistence or decay rates were also extracted to evaluate the extent to which studies investigated the reliability of FIB as temporal indicators. Information on sediment mixing processes such as bioturbation was also extracted where reported, as these factors may influence the vertical distribution of the bacteria. Data related to surface sediments or sediment cores from a river section for example, which may have also been reported in the studies, were not included in this review.

## 3 Results

### 3.1 Study site characteristics

**Fig. 2:**
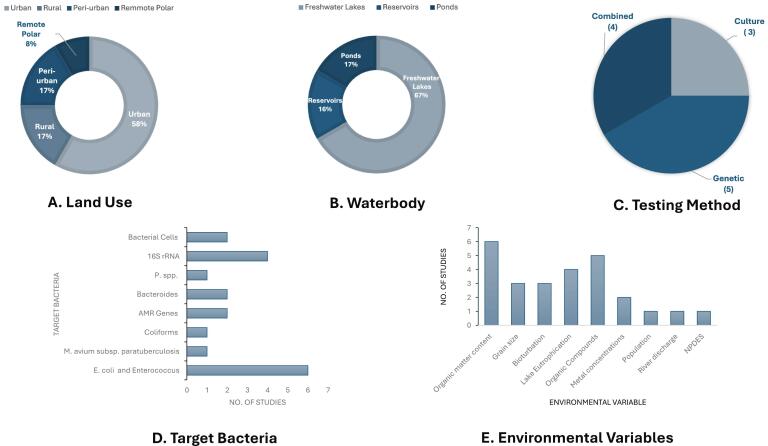
Summary of the study environment context, analytical methods and possible influencing environmental variables across the included studies (*n* = 11). A. Land use type surrounding study site. B. Aquatic system classification of each study. C. The number of studies which employed genetic, culture or a combination of both testing methods. D. Types of target bacteria employed in the study. E. Other possible environmental variables which could be influencing the distribution of bacteria throughout the sediment cores and the number of studies which they appeared in.

A total of 12 studies were identified and subject to data extraction (Fig. 1). A summary of the studies is outlined in Table S2 (Supplemental Material). All identified articles were published since the year 2000, with the majority of studies (75 %) being published between the years 2010–2019. Geographically, the majority of studies were based in Europe (*n* = 9/12, 75 %). In the context of local environment or ‘setting,’ the primary land use type among studies was ‘urban’ accounting for 7/12 (58 %) studies, followed by ‘rural’ and ‘peri-urban’ classifications and one study which could be classified as a remote polar environment (Fig. 2).

In terms of system classification, a total of eight (67 %) studies focused on freshwater lake environments one which was an Antarctic Lake, two (17 %) studies focused on reservoirs, while the remaining two studies were focused on pond environments, one of which was specifically a forest hollow/closed depression (Fig. 2). Half of the studies, 50 % (6/12), reported that the freshwater source was used for human consumption. It was found that 67 % (8/12) of researchers collected cores from more than one sampling location. The remaining studies sampled cores from just one location, with one investigation sampling multiple cores from the same location to perform a persistence study [38]. In the other three studies, there was only one lake sediment core retrieved.

### 3.2 Study design

#### 3.2.1 Sediment coring and chronology

Overall, reviewed investigations represent a total of 35 collected freshwater lake sediment cores, with the maximum number of cores extracted in a single study being six cores [51]. These sediment cores varied in length from 9 cm to 130 cm and in diameter from 6 cm to 14 cm. The average depth of the water from which these cores were extracted was 54.3 m. The deepest core was collected from a water depth of 304 m in Lake Geneva [52], while the shallowest extraction occurred at 20 m water depth [53]. The water depth at which the cores were collected was not specified in six of these studies.

Geochronological dating techniques were used in nine of the 12 studies reviewed. The chronological dating techniques used in these studies include ^137^Cs, ^14^C, ^210^Pb, ^226^Ra, ^241^Am, and ^7^Be. Of the methods used, ^137^Cs was the most common, employed in four of the five studies that conducted original dating of cores. Among these, one study reported discontinuous sedimentation in the core's chronology. Four of the eight studies dated only portions of the cores. Four studies utilised previously dated cores from the same locations as a reference for dating newly collected cores.

#### 3.2.2 FIB analysis

Half of the studies (6/12) used *E. coli* and *Enterococcus*, which were the most common faecal indicator bacteria used to track organic pollution in lake sediments. Fig. 2 summarises the testing method employed among all reviewed investigations, with five opting for genetic testing methods, three studies relying on culture-based methods, and four studies combining both methods. While most studies employed well-established FIB (e.g., *E. coli, Enterococcus*), one study instead used the zoogenic *Mycobacterium avium* subspecies *paratuberculosis* to trace organic pollution. Overall, only two investigations assessed antimicrobial resistance in the cores.

Additionally, four out of the 12 studies included additional methods of measuring faecal pollution. These studies assessed faecal sterols and stanols in sediment, as a biomarker for microorganism activity using ATP tests, pyrosequencing, bioinformatics, and bacterial community genetic fingerprinting without relating it to faecal pollution. These methods were beyond the focus of this review as they were not specific to FIB, and thus, did not meet the inclusion criteria.

Beyond FIB analyses and aiming to gain insights into the depositional (sediment) environment, several investigations incorporated the characterisation of various physico-chemical and biological parameters. These included sediment or particle grain size, which was measured in three of the 12 studies. Metals in the sediment were analysed in four studies, with mercury being the most frequently examined metal. Physicochemical parameters such as organic carbon, nitrogen content, and total phosphorus were measured in eight studies; one of these studies also included carbon and nitrogen isotope ratio measurements. Organic content was assessed through loss-on-ignition in six studies. Environmental or anthropogenic data were collected in two investigations.

### 3.3 Faecal Indicator abundance, temporality and distribution in sediment cores

A summary of bacterial abundance and proliferation in the analysed sediment cores, included the depth of detection at which the increased abundance of bacteria/ genes occurred and corresponding timeframes, is presented in Table 4. Ten out of 12 investigations analysed FIB in multiple lake cores, while two studies relied on a single core (Table 4). In terms of spatial trends, six studies reported that the highest concentrations of both FIB and antibiotic resistance genes (ARGs) were found in the most polluted areas of the lakes. The remaining six studies either did not observe this correspondence or did not assess it due to the use of only one core per site. Temporal trends were also identified in several studies. Higher bacterial counts were found towards the middle and top of the cores, with bacterial abundance generally decreasing with depth in nine out of the 12 studies. Additionally in cores extracted near point sources of pollution, spikes in bacteria numbers were observed throughout the core with the distribution of bacteria down the core being closely linked to timing of pollution events in the waterbodies (7/12). For example, all core analyses from the Lake Geneva studies showed an increase in bacterial and gene abundance after approximately 1970. Two additional studies identified distinct temporal thresholds associated with elevated bacterial levels. One study [54] reported stable concentrations of *Enterococcus* during 1760–1860 and 1910–2003, but noted increases in *E. coli* in 1849 and 1890. Another study [55] found increased abundance of *Bacteroidales* dating to between 2150 and 1650 calibrated years before present (cal BP). Only one study discussed bacterial decay rates explicitly by investigating *E. coli* and *Enterococcus* survival over a 90-day period. Overall, both the depths and estimated ages at which increases in bacterial, or gene abundance occurred varied between studies, reflecting heterogeneity in site history, pollution inputs and core resolution.

**Table 4:** Summary of the trends of bacteria in the cores of each study and the depth and year the increasing abundance of bacteria/ genes occurs (Year occurrence cal BP refers too calendar years before present). NA applied to data which is not applicable; NR applied to data which is not reported.

Study	Multiple lake cores analysed for bacteria present	Core with highest bacteria / ARGS in most polluted area	Increasing Bacterial/ Gene Abundance/ Diversity Depth Occurrence	Increasing Bacterial/ Gene Abundance/ Diversity Year Occurrence
Pickup et al., 2005	No	NR	NR	NR
Li et al., 2006	No	NR	21–45 cm	NR
Haller et al., 2009	Yes	NA	0–2 cm	NR
Vane et al., 2010	Yes	Yes	0–10 cm	NR
Thevenon et al., 2011b	Yes	Yes	0–18 cm	After ca. 1970
Thevenon et al., 2012a	Yes	Yes	Ca. 24 cm	After ca. 1970
Thevenon et al., 2012b	Yes	Yes	0–20 cm	After ca. 1970
Sauvain et al., 2014	Yes	Yes	0–3 cm	NR
Devarajan et al., 2015	Yes	Yes	35–40 cm	Ca. 1970s
Etienne et al., 2015	Yes	NR	283–297 cm	2150–1650 cal BP
Brooks et al., 2016b	Yes	NA (all from same location)	NR	**ENT:** 1760–1860 AND 1910–2003 **EC:** 1890 AND 1849
Sun et al., 2021	Yes	NA	15–25 cm	NR

### 3.4 Potential pollution inputs and influencing factors

Wastewater treatment plant effluent or sewage pollution is stated as the most likely pollution source in nine out of the 12 studies. In turn, livestock in the catchment was described as the probable pollution source in two and one study stated the likely pollution source as penguin guano. A total of six investigations report spikes in bacteria concentration throughout the sediment core can be linked to known or specific pollution events in time. However, five of the articles were not definitive in linking fluctuations in FIB concentrations to an eliciting event and one study could not link the distribution of bacteria in the core to the timing of pollution input events. As well as this Fig. 2 displays additional environmental variables that may influence bacterial distribution in the sediment cores such as organic compounds or nutrients, metals present and grain size. Eutrophication, which is a symptom of pollution due to elevated levels of nutrients in the aquatic environment, was an influencing factor of bacterial distribution in four studies. Bioturbation was found to influence bacteria distribution through the core in two studies.

## 4 Discussion

Our study highlights a consistent reliance on *E. coli* and *Enterococcus* as the primary FIB for assessing faecal contamination in lakes, with over half of the studies employing these bacteria. This is likely due to their established role in water quality monitoring in surface and groundwater systems [56,57]. *E. coli* is strongly connected to pollution of faecal origin and is a widely used indicator organism in water quality assessment in freshwater environments. However, there is a growing body of evidence to suggest that it can be part of the naturally occurring micro-biota of soils and it is important to take this into account in pollution studies [[58], [59], [60]]. *Enterococcus* is also a widely used faecal indicator for water quality assessments, particularly in marine and recreational waters, due to its high salt tolerance [61]. However, it is also applied in freshwater monitoring. Although it has to be noted that not all enterococci are exclusively from faecal origin [60]. Perhaps this may be the rationale for testing *Enterococcus* together with *E. coli* to confirm faecal pollution, and why one study measured *E. faecium* and *E. faecalis,* which are specifically found in human faecal matter. Both of these bacterial species can be cultured and enumerated through inexpensive methods. Although research has focused on the identification of bacteria species that may indicate organic pollution in freshwater lake environment sediment cores, it is clear from this review that inadequate research exists on the decay rates of faecal related bacteria in lake sediments. More specifically the long-term decay rates in these sediment cores are unknown. Within this review, only one study investigated the survival of *E. coli* and *Enterococcus* in the core over a 90 day period [38]. Similarly, only one other study identified unknown decay rates of faecal bacteria in cores as a limitation in contamination investigations [62].

The limited number of studies included in this review (*n* = 12) highlights that the use of isolated FIBs in sediment cores to study faecal pollution in freshwater environments is relatively uncommon and under studied. A key challenge in using FIBs to trace organic pollution in lake cores lies in their limited preservation. Unlike traditional paleolimnological proxies such as diatoms or pollen, FIB do not fossilise and can degrade overtime [40]. However, several studies have demonstrated that FIB or their genetic material (e.g., DNA, ARGs) can persist long enough to allow for retrospective detection and analysis. These findings suggest that while preservation is not guaranteed, it is possible, making time-sensitive analysis and contextual interpretation essential. Future research should explore the long-term decay rates of these bacteria and assess whether genetic or culture-based methods yield more reliable results. Integrating additional proxies, such as faecal stanols and sterols, may also help to validate and strengthen this approach.

The presence of antibiotic resistance (AMR) in the sediment cores was investigated in only two of the studies in this review [63,64], which highlights a significant knowledge gap and an important avenue for future research. Both studies focused on Lake Geneva (Switzerland) and used molecular methods to detect ARGs in lacustrine sediments. Thevenon [63] demonstrated a marked increase in multiple antibiotic resistant (MAR) bacteria and ARGs such as blaTEM, aadA, tetA and cmlA, following the onset of cultural eutrophication and effluent discharge from the WWTP (ca. 1970) in Lake Geneva. Devarajan [64] reported the accumulation of clinically relevant ARGs, including genes encoding resistance to β-lactams, alongside elevated bacterial loads and trace metal concentrations in lake sediments. These findings suggest that lake sediments may act as long-term reservoirs for AMR, influenced by anthropogenic pressures such as urban runoff and wastewater discharge. The United Nations Environment Programme listed the role of the natural environment in resistance development as an emerging issue of environmental concern [65]. Thus, understanding how AMR has evolved or persisted in the environment over time is crucial. Gaining a deeper insight into these dynamics will be essential for informing policies to mitigate this growing public health risk.

### 4.1 Spatial pattern of FIB distribution

A clear trend emerged in studies analysing multiple lake cores, where in most cases, higher bacterial concentrations were found close to pollution sources. This pattern was particularly evident in the five studies that focused on Lake Geneva, where proximity to WWTP effluent correlated with higher bacterial concentrations and other environmental factors, such as metal pollution and pharmaceuticals contamination [33,66]. The extensive research on Lake Geneva highlights how intensively/well-studied waterbodies can offer rich datasets for identifying pollution patterns, providing useful models for future research. Conversely, multiple articles did not report if the cores were located in the most polluted area [38,[53], [54], [55],^67^]. This may be due to limitations in the data, such as sediment cores being collected from a single location or only one core being analysed. Additionally, challenges in identifying pollution sources may arise, particularly when pollution originates from diffuse non-point sources, such as agricultural activities within the catchment area.

This highlights the necessity for comprehensive research focused on areas affected by pollution to accurately identify the source of pollution (point source and diffuse). Identifying point sources of pollution, such as effluent pipes or sewage discharge from WWTP, is relatively straightforward. The task of identifying managing and controlling diffuse pollution such as water contamination by livestock is considerably more difficult [68].

### 4.2 Vertical FIB distribution and implications for reconstructions

This review indicates a trend where higher bacterial counts are found towards the middle and top layers of the core, with bacteria abundance generally decreasing with depth (9/12). However, in cores extracted near point sources of pollution, spikes in bacteria numbers were observed throughout the core. This pattern can be linked to pollution input events and tends to be associated with elevated levels of organic matter or metals which are often discharged in unison from the WWTP and from agricultural/domestic wastewater [69,70].

The distribution of bacteria down the core could be closely linked to the timing of pollution events in the waterbody for the majority of studies in this review (6/12). This was evident in studies which had cores with age-depth chronologies and clear histories of pollution events in the waterbody such as implementation and construction maintenance of wastewater treatment plants, increasing population of the watershed, when and if the waterbody was considered eutrophic as well as the agricultural practices of the area or livestock abundance. Increased abundances of these bacteria were even evidenced to occur before the regulations regarding wastewater treatment, septic tanks and agricultural practices for example were introduced [71]. However, there were two studies specifically from Lake Geneva which linked bacteria distribution in the core with the impact of eutrophication in the 1970s and 1980s rather than evidencing the implementation of the WWTP effluent pipe in 1964 [63,72]. As well as two other Lake Geneva studies which stated both eutrophication of the lake and the influence of the WWTP as the reasoning for an increased bacterial load [52,64]. A possible limitation identified due to this discovery was bioturbation which needs to be taken into account in future studies. Overall, for Lake Geneva studies the FIB, MAR and antibiotic-resistant bacteria in the core from the most contaminated area of the lake, which is influenced by the WWTP effluent, were found to dramatically increase in sediments deposited after 1970 when the lake was considered eutrophic. Furthermore, the depths at which the increasing gene/ bacterial abundances were observed in the cores varied between studies.

Conversely, other studies were classified as not following or not definitively following a pollution pattern [38,51,53,67,73,74]. Issues with these studies were mainly due to uncertain or undated core chronologies, the possibility of bioturbation or slumping occurring, or a diffuse pollution source which makes it more difficult to find the exact location or timing for pollution input. Another issue which was identified in one of these papers was that it did not employ a widely used FIB and the exact amount of this bacteria (*Mycobacterium avium* subsp. *paratuberculosis*) was not measured [53]. Rather, samples were categorised as PCR positive or negative to indicate presence/absence. Notably the type of indicator bacteria used and the method of measurement of bacteria is important to distinguish trends. For instance *E. coli* and *Enterococcus* are well established FIB species [75] as opposed to *M. avium* subsp. *paratuberculosis* used in this paper. There are also clear advantages for using quantitative methods to measure bacterial abundance throughout sediment cores, as they provide a more precise understanding of the correlations between bacterial distribution and faecal contamination. However, to draw more robust conclusions across studies, it is essential that investigations use consistent faecal indicator bacteria such as *E. coli* and *Enterococcus* as well as standardised quantification methods to limit complications regarding comparisons between studies.

Overall, this review identified several studies which reported that maximum bacteria in the core often coincides with the estimated timing of the pollution which occurred. However, this pattern was not consistently observed across all investigations. In many cases, limitations such as undated or poorly dated cores, unclear pollution histories or inconsistent use of indicator bacteria and quantification methods impeded the ability to draw definitive conclusions. In the studies where a temporal relationship between bacterial abundance and known pollution events was observed, the findings highlight the importance of using well-dated sediment cores, detailed historical pollution records and established bacterial indicators alongside quantitative techniques to reconstruct past pollution events. There was a significant limitation identified in this review regarding the limited geographical variation in studies. The majority of available data is from a single site, Lake Geneva. To address this limitation and gaps in the literature, future research could focus on completing similar studies across a wide variety of lake types and locations.

### 4.3 Important environmental variables influencing bacterial distribution

Various environmental variables have been shown to influence the distribution and survival of bacteria in lake sediments including organic matter, metals and grain size.

As previously mentioned, three studies attribute the vertical distribution of pathogens throughout the sediment cores to increased inputs of organic-rich material, which in turn led to eutrophication of the lake [63,64,72,74]. Organic-rich material often includes elevated levels of nutrients such as phosphorus and nitrogen [32]. For example, in Lake Geneva, rising concentrations of phosphorus and nitrogen were observed during the period leading up to and during eutrophication, contributing to shifts in microbial dynamics within the sediment record [71]. According to Li (2021) sediments of lakes which have high organic content aid in the survival of *E. coli* at lower temperatures, while Haller (2009) hypothesises that high organic content in the sediment may also lead to a higher rate of FIB adsorption. Thevenon (2011b) attributes the increasing bacterial abundance with time to the high sedimentary organic matter content and states that it likely came from sustained anthropogenic organic pollutant inputs from the WWTP. Brooks (2016) states that organic materials provided in sediment may protect cells from sunlight and potential predation. In contrast to this Etienne (2015) and Sun (2021) state that the distribution of bacteria in the cores had a significant negative correlation with organic matter and organic nutrients. Although unproven, this may stem from challenges in identifying and tracking pollution over time, particularly as the source is diffuse, making it harder to establish links between bacterial distribution and this type of pollution. Hence further research on this topic is essential.

There was an association of the occurrence of FIB with the presence of metals such as Cr, Cu, Zn, As, Cd, Pb, Hg, Fe, Ag in the sediment core. There was also a strong correlation observed between endospore-forming bacteria and high total particulate trace metals (TM_part_) concentrations, and Sauvain (2014) states that the mechanism explaining the relationship between endospore-forming bacteria and TM_part_ sorption requires more research. High metal contamination which is the case in the sediment of Lake Geneva can create an important reserve for bacteria ARGs and multiple antibiotic-resistant bacteria (MARs) [63]. This may indicate that the bacterial load increases with increasing metal levels and this co-selection might influence the selection of ARGs within the lake, with Devarajan (2015) stating that metals can co-select for ARGs. It has also been stated that heavy metals may increase antibiotic resistance due to indirect selection [76].

A high proportion of fine particles may play a role in the FIB adsorption in the sediment profile [38,54,64]. It was reported that *Enterococcus* concentrations were significantly correlated with river discharge and it was suggested that the attachment of *Enterococcus* to suspended particles may aid in their movement to the benthos of lakes [54].

Devarajan (2015) noted that metals, bacterial marker genes and ARGs increase as the organic matter content in the lake increases as well as a correlation being found between organic content in sediment specifically C_org_, N_org_ and trace metal concentrations [51]. This may be evidence for all these variables being discharged from the same source into the lake, as wastewater effluent can contain large amounts of metals, nutrients, organic matter and bacteria which can be deposited in surrounding sediment [69]. The studies did not review measurements of contaminants that were discharged from the wastewater treatment plant and future studies could look at this knowledge gap.

The extensive use of antibiotics was stated as a driving factor for the distribution of antibiotic-resistant genes in the sediment [63,64]. It was reported that resistant genes were present in many sediment samples and suggested that this may be due to the bacterial community acquiring resistant genes mainly from human activities and possibly from horizontal gene transfer (HGT) between bacteria [63]. However, Thevenon et al. (2012a) questions whether this occurs before widespread antibiotic dissemination, e.g. in hospitals or the environment, and the role of commensal bacteria such as from soils or sediments as recipients or donors of DNA still needs further investigation. It was also reported that preindustrial sediments can be a reservoir for antibiotic-resistant genes due to the presence of the aadA (streptomycin and spectomycin) resistance gene found in the sediments which were deposited before the WWTP effluent water table [63]. This indicates that the extensive use of antibiotics in human medicine, agriculture and aquaculture for more than a century and suggests ARGs can be considered as an emerging contaminant in freshwater sediments for this period [63,[77], [78], [79]].

It is evident from the aforementioned studies in this review that there has been a robust amount of research carried out on the influence environmental variables can have on the distribution of bacteria in sediment cores with studies looking at the influence of organic matter content to metals. However, no study investigated all of these the variables comprehensively. Future studies could focus on the influence of the identified variables in one individual study to fully comprehend the differing impacts environmental variables can have on the distribution of bacteria throughout sediment cores.

## 5 Conclusion

This scoping literature review demonstrates that bacteria identified in lake sediment cores can effectively track organic pollution. Well-established FIB species such as *E. coli* or *Enterococci* provide the most reliable method of tracing faecal pollution. Their presence is generally indicative of faecal contamination, as these bacteria are not as commonly found in the environment without a pollution source as other bacteria. This allows for confident attribution of observed bacteria to pollution inputs. To enhance the robustness of future research, it is advisable to employ a combination of proxies, including faecal stanols and sterols, to confirm that bacterial distributions are indeed related to faecal pollution.

The importance of accurately dated sediment cores is essential for correlating bacterial distribution with pollution events through time. A comprehensive understanding of the historical context of pollution sources is also essential to accurately interpret the distribution patterns. While bacteria can be used to track faecal pollution through time in lake environments, various factors such as organic matter, metals presence, and grain size can influence their persistence in lake environments. It is important to account for these different environmental factors, which can affect bacterial viability and detectability. Given that wastewater effluent contains high concentrations of metals, nutrients, and organic matter, these components may correlate strongly with bacterial presence.

Further research is needed to validate the use of bacteria in lake sediment cores as a method of tracking faecal pollution and to establish its reliability. Future studies should also explore the presence of antimicrobial resistance in lake sediment cores and how resistance profiles have evolved over time. A focus should be placed on developing a standardised procedure which could be used globally to identify at-risk areas. This would allow effective monitoring to be carried out by local authorities. This critical evidence base could be used to inform policymakers when implementing current and future policies relating to environmental pollution and human health.

## CRediT authorship contribution statement

**Julie O'Donovan:** Writing – original draft, Visualization, Methodology, Investigation, Formal analysis, Conceptualization. **Jean O'Dwyer:** Writing – review & editing, Supervision, Funding acquisition, Conceptualization. **Michelle McKeown:** Writing – review & editing, Supervision. **Aaron Potito:** Writing – review & editing, Supervision, Funding acquisition, Conceptualization.

## Declaration of competing interest

The authors declare that they have no known competing financial interests or personal relationships that could have appeared to influence the work reported in this paper.

## Data Availability

Data will be made available on request.

## Appendix A Supplementary data

Database search terms which were employed Image 1

Data extraction spreadsheet Image 2
