# Supplementary material for: Bacteria isolates from Lake sediments as a promising proxy for temporally tracking organic pollution: A global review
Source: One Health. 2025 Aug 30;21:101184. doi: 10.1016/j.onehlt.2025.101184 (PMC12446193; doi:10.1016/j.onehlt.2025.101184)
Supplement: Supplementary file 1 — Database search terms which were employed [file mmc1.pdf]

Table S1: Database search terms which were employed

Mock searches:

Database	Search	Results
**Scopus**	(TITLE-ABS-KEY ("Pond Core" OR "Lake Core" OR "Lagoon Core" OR "Reservoir Core" OR "Freshwater Core" OR "Inland water core" OR "sediment Core" OR "Lake Core" OR "Lentic Core" OR "Lake Substrate Core" OR "Lake Sediment Core" OR "Lake Core" OR "Lake Transect" OR "Lake Stratigraphy" OR "Lentic Core") AND TITLE-ABS-KEY ("fecal" OR "faecal" OR "fecal indicator" OR "faecal indicator" OR "fecal indicator organism" OR "faecal indicator organism" OR "FIO" OR "coliform" OR "coliforms" OR "total coliforms" OR "total coliform" OR "fecal indicator bacteria" OR "faecal indicator bacteria" OR "FIB" OR "coliphages" OR "coliphage" OR "microbial indicator" OR "gram-negative bacteria" OR "gram-positive bacteria" OR "bacteria" OR "microbial" OR "E.coli" OR "E. coli" OR "escherichia coli" OR "enteric bacteria" OR "enterococcus" OR "enterococci" OR "microbe") AND TITLE-ABS-KEY (contamina* OR pollut* OR presence OR incidence OR preserv* OR dissemination OR dispersal))	725
**Web of Science**	((TS=("Pond Core" OR "Lake Core" OR "Lagoon Core" OR "Reservoir Core" OR "Freshwater Core" OR "Inland water core" OR "sediment Core" OR "Lake Core" OR "Lentic Core" OR "Lake Substrate Core" OR "Lake Sediment Core" OR "Lake Core" OR "Lake Transect" OR "Lake Stratigraphy" OR "Lentic Core")) AND TS=("fecal" OR "faecal" OR "fecal indicator" OR "faecal indicator" OR "fecal indicator organism" OR "faecal indicator organism" OR "FIO" OR "coliform" OR "coliforms" OR "total coliforms" OR "total coliform" OR "fecal indicator bacteria" OR "faecal indicator bacteria" OR "FIB" OR "coliphages" OR "coliphage" OR "microbial indicator" OR "gram-negative bacteria" OR "gram-positive bacteria" OR "bacteria" OR "microbial" OR "E.coli" OR "E. coli" OR "escherichia coli" OR "enteric bacteria" OR "enterococcus" OR "enterococci" OR "microbe")) AND TS=(contamina* OR pollut* OR presence OR incidence OR preserv* OR dissemination OR dispersal)	199
